# Supplementary material for: Dysglycemia is associated with Mycobacterium tuberculosis lineages in tuberculosis patients of North Lima—Peru
Source: PLoS One. 2021 Jan 28;16(1):e0243184. doi: 10.1371/journal.pone.0243184 (PMC7843012; doi:10.1371/journal.pone.0243184)
Supplement: S1 Table — (DOCX) [file pone.0243184.s001.docx]

**S1 Table.** Baseline epidemiological and clinical characteristics of the study population.

| Characteristics | Total | TB-DM | TB-pDM | TB | p-value* |
| --- | --- | --- | --- | --- | --- |
|  | (N=112) | (n=17) | (n=33) | (n=62) |  |
| TB symptoms |  |  |  |  |  |
| Dyspnea | 70 (62.5) | 12 (70.6) | 16 (48.5) | 42 (67.7) | 0.62 |
| Blood in sputum | 48 (42.9) | 8 (47.1) | 12 (36.4) | 28 (45.2) | 0.85 |
| Night sweats | 69 (61.6) | 11 (64.7) | 19 (57.6) | 39 (62.9) | 0.94 |
| Drug resistance |  |  |  |  |  |
| MDR TB | 9 (12.3) | 2 (18.2) | 3 (13) | 4 (10.3) | 0.48 |
| Non-MDR TB | 19 (26.0) | 3 (27.3) | 8 (34.8) | 8 (20.5) | 0.41 |
| DM symptoms |  |  |  |  |  |
| Polydipsia | 54 (48.2) | 9 (52.9) | 17 (51.5) | 28 (45.2) | 0.49 |
| Polyuria | 43 (38.4) | 8 (47.1) | 12 (36.4) | 23 (37.1) | 0.55 |
| Fatigue | 89 (79.5) | 13 (76.5) | 25 (75.8) | 51 (82.3) | 0.48 |
| Delayed wound healing | 16 (14.4) | 2 (11.8) | 9 (28.1) | 5 (8.1) | 0.21 |
| Dysglycemic control drug use |  |  |  |  |  |
| Metformin | 11 (9.8) | 11 (64.7) | 0 (0) | 0 (0) |  |
| Insulin | 1 (0.9) | 1 (5.9) | 0 (0) | 0 (0) |  |
| ^·^Other drugs | 5 (4.5) | 5 (29.4) | 0 (0) | 0 (0) |  |
| Comorbidities |  |  |  |  |  |
| Alcoholism | 57 (51.4) | 4 (23.5) | 22 (68.8) | 31 (50) | 0.32 |
| Smoking | 24 (21.6) | 4 (23.5) | 8 (25.0) | 12 (19.4) | 0.59 |

Data represent no. (%). TB=pulmonary tuberculosis, DM=diabetes mellitus, TB-DM=pulmonary tuberculosis and diabetes mellitus comorbidity, TB-pDM=pulmonary tuberculosis and prediabetes comorbidity at screening, BMI=body mass index.

**^·^**Metformin plus glibenclamide or metformin plus insulin or no drug use.
