# Supplementary material for: Dysglycemia is associated with Mycobacterium tuberculosis lineages in tuberculosis patients of North Lima—Peru
Source: PLoS One. 2021 Jan 28;16(1):e0243184. doi: 10.1371/journal.pone.0243184 (PMC7843012; doi:10.1371/journal.pone.0243184)
Supplement: S2 Table — (DOCX) [file pone.0243184.s002.docx]

**S2 Table.** Agreement analysis between 24-MIRU-VNTR and whole genome sequencing among 86 *M. tuberculosis* strains.

| **Method** | **Whole Genome Sequencing**  **Concordance (%)** | | | | | |
| --- | --- | --- | --- | --- | --- | --- |
| **24 MIRU-VNTR** | Beijing  (n, %) | LAM  (n, %) | Haarlem  (n, %) | **Others  (n, %) | Total | Kappa coefficient (k) |
| Beijing (n, %) | 13 (92.9) | 0 (0) | 0 (0) | **1 (5)^*^** | 14 (16.3) | 0.7 |
| LAM (n, %) | 0 (0) | 28 (87.5) | 0 (0) | 0 (0) | 28 (32.6) |  |
| Haarlem (n, %) | 0 (0) | 0 (0) | 19 (95) | **9 (45)^*^** | 28 (32.6) |  |
| **Others (n, %) | **1 (7.1)^*^** | **4 (12.5)^*^** | **1 (5)^*^** | 10 (50) | 16 (18.6) |  |
| Total | 14 (100) | 32 (100) | 20 (100) | 20 (100) | 86 (100) |  |

MIRU-VNTR, Mycobacterial Interspersed Repetitive Unit Variable Number Tandem Repeat.

*In black, discordant results of *M. tuberculosis* lineages assignment by both molecular techniques.

**Others *M. tuberculosis* lineages: S, X, Cameroon, Ghana, Ugandal, mixed strains.
